# Assessing the impact of common sample preparation strategies for single particle ICP-MS regarding recovery and size distribution of natural single particles

**DOI:** 10.1039/d5ja00170f

**Published:** 2025-08-20

**Authors:** Lhiam Paton, Sandra Kiesel, Grit Steinhoefel, Matthias Elinkmann, Thebny Thaise Moro, Raquel Gonzalez de Vega, Pascal Bohleber, David Clases

**Affiliations:** a Trace Element Speciation Laboratory (TESLA), Institute for Analytical Chemistry, University of Graz 8010 Graz Austria lhiam.paton@uni-graz.at; b Alfred Wegener Institute Helmholtz Centre for Polar and Marine Research 27570 Bremerhaven Germany; c Department of Geosciences, Goethe University Frankfurt Am Main 60438 Frankfurt Am Main Germany; d NanoMicroLab, Institute for Analytical Chemistry, University of Graz 8010 Graz Austria

## Abstract

Particles on the nano- and micro-scale are produced in a wide range of natural and anthropogenic processes and play a significant role in the biogeochemical cycling of major and trace elements in the environment. Single particle inductively coupled plasma – mass spectrometry (SP ICP-MS) is quickly becoming one of the premier techniques for analysis of nano- or micro-entities. However, SP ICP-MS analysis requires dilute aqueous solutions, free from large particles that could cause blockages. For environmentally relevant samples, like soil extracts, this typically calls for sample clean up. Sample preparation strategies like syringe filtration or ultra-centrifugation are regularly applied to handle complex matrices. The aim of this article is to examine the influence of common preparative strategies on the analysis of both naturally formed and synthetic nanoparticles in complex matrices. To achieve this, water extracts of mineral and sediment standards were spiked with Au nanoparticles and a variety of chemical and physical approaches were investigated to identify which strategies provide the best route to accurately quantifying particle numbers, masses and sizes. In a vast majority of cases, at least 90% of the detectable particles were lost for both particle types whenever filtration or centrifugation was applied. The addition of surfactants like Triton X-100 proved to promote relative particle recoveries of up to 30% for spiked Au particles but the extracted Fe-containing particles continued to have losses of up to 99%. Therefore, common sample preparation strategies are directly impeding the possibilities for quantitative particle analysis by SP ICP-MS. Furthermore, commonly used nanoparticles like Au do not necessarily reflect the reality of nano- and microparticles found in the environment. It is apparent that for SP ICP-MS to become a useful, quantitative method for environmental analysis there must be a high degree of care taken in the collection and preparation stages of analysis.

## Abstract 2

SP ICP-MS identified that sample preparation approaches (*i.e.*, filtration) resulted in a significant loss of detected particles extracted from environmental samples.

## Graphical abstract

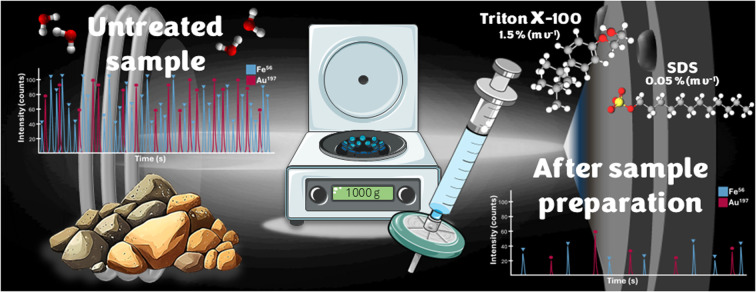

## Introduction

Nano- and microparticles are cycled at high rates across the Earth's surface and take active roles in countless mechanisms and processes in the environment.^1^ Besides natural entities, incidental anthropogenic particles (*e.g.*, microplastics^2^) are increasingly being released into the environment and pinpointing and understanding them remains a vast challenge. Furthermore, particles are frequently manufactured (engineered particles) for industrial, medical or consumer applications and these also need to be controlled in both composition as well as size distribution. Different analytical techniques, including electron microscopy (EM),^3^ dynamic light scattering (DLS), nanoparticle tracking analysis (NTA)^4^ or single particle inductively coupled plasma – mass spectrometry (SP ICP-MS)^5^ have been developed to study particles. Among these, SP ICP-MS is gaining momentum to provide a detailed perspective on individual particles in a one-by-one fashion while setting new benchmarks for the characterisation of particles.^5,6^ It provides opportunities to extract various particulate parameters, like particle number concentrations (PNC), isotopic information, masses and sizes from complex samples while coping with difficult matrices for particles with dimensions ranging across the nanoscale and lower microscale. Its operation paradigm is based on the randomised, individual introduction of particles into a hot argon plasma, where particles are atomised, and the resulting atoms are ionised.^5,7^ Consequently, each particle produces an ion cloud which can be extracted for mass spectrometry. The most applied mass analyser for SP ICP-MS is the quadrupole, which is applicable to study specific particulate elements, but it is limited when aiming to characterise particle composition and for screening unknown samples for particulate elements.^8,9^ This is anchored in the relatively long settling and scanning times of quadrupoles, which are longer than the transit times of individual ion clouds. This issue can be overcome when using time-of-flight technology (SP ICP-TOFMS).^8,10^ SP ICP-TOFMS provides an unprecedented level of detail and gains information on the composition of individual particles. Consequently, it enables their clustering into common groups and determination of group-specific number concentrations as well as mass and size distributions. This has the potential to be particularly impactful for the analysis of environmental samples, which typically contain complex matrices (*i.e.*, soil or river water) with a wide range of polymetallic particles which cannot be differentiated by SP ICP-QMS.^11^ While this promises new avenues to characterise environmental samples, we still lack a fundamental understanding of how particles behave during sampling, storage, and preparation and how analytical strategies and protocols need to be designed to enable reliable and reproducible analysis.

In this study, we aim to highlight common sample preparation strategies and provide insights into critical effects that apply to natural and synthetic particles. Adequate preparation strategies need to produce a suitable sample which can be effectively analysed^12–14^ while ensuring high recoveries, preserving particle integrity and minimising size or species bias. This is a difficult task, and most strategies aim to minimise sample manipulation and favour “dilute and shoot” methods. However, even these methods bear the potential to induce changes in ionic strength and as such particle stability.^9^ Other strategies apply additional filtration,^15–17^ centrifugation^18–20^ and/or additives, which however need to be evaluated carefully as they may inadvertently result in an irreversible transformation of the particulate analytes.

The use of syringe filters is common practice for the clean-up of environmental samples^6^ prior to any subsequent particle analysis and aims to provide a homogenous sample whilst avoiding instrumental blockages. Syringe filters are developed with specific particle size cut-offs, which, in principle, preserve the integrity of particles that are smaller in diameter than the designated cut-off. However, the physical and chemical properties of nanoparticles vary greatly with environmental matrices, composition, shape and size.^21^ This is particularly notable, for environmental samples, where changes in conditions such as salinity or pH may alter the interactions between the filter material and the analyte, therefore, affecting measurement accuracy.

The effects of sample preparation have been investigated in recent years^22,23^ and mostly focussed on means to achieve higher particle recoveries. Typically, well characterised and simple, single-phase particles such as Au or Ag are chosen to investigate effects from sample preparation in aqueous matrices. However, it is worth mentioning that these model particles have very different physical and chemical properties compared to natural occurring species such as surface reactivity, shape or chemical compositions. The identification and characterisation of naturally occurring nanoparticles present in the environment is of high interest and here we raise questions on how transferable insights from the investigation of synthesised model particles are when compared to those formed through natural processes.

As such, the aim of this study is to present an example case study to highlight the difficulties associated with the analysis of samples collected in an environmental context (*i.e.*, soil samples) where the particle composition may be entirely unknown. This article includes an evaluation of several sample preparation strategies including physical (*i.e.*, filtration, centrifugation) and chemical (*i.e.*, stabilisation through surfactant addition) manipulation. To achieve these objectives, we suspended particles from different mineral and marine sediment standard materials as proxies for different environments and applications and focused on the behaviour of extractable Fe-containing particles; which was found to be a common analyte across sample types. Furthermore, nanoparticle standards (Au, 100 nm) were spiked into every sample to compare relative recoveries and as a benchmark to evaluate the impact of specific sample preparation operations regarding number and size distributions. Furthermore, this enabled an assessment whether synthesised nanospheres can act as good analogues for the behaviour of naturally formed nanoparticles. Finally, we discuss how the handling of environmental samples may impact particle species differently, which has major implications for multi-element approaches (*i.e.*, application of SP ICP-TOFMS).

## Methodology

### Chemicals

The rock and mineral standards which were used for this study are presented in Table 1. 100 nm ± 8 nm Au nanoparticles were used for calibration (nanoComposix, USA, citrate stabilised), prior to analysis they were stored at 4 °C. TraceCERT elemental standards for ICP-MS were purchased from Merck (Vienna, Austria, 1000 mg L^−1^, 12% HNO_3_) and were diluted immediately prior to analysis in ultra-pure water (MilliQ, 18.2 MΩ cm, Millipore, Bedford, USA). The following chemicals were employed as additives for the purpose of investigating particle recoveries: Triton X-100 (Fisher Scientific, USA), sodium dodecyl sulphate (SDS, Fisher Scientific, USA), tetrasodium pyrophosphate (98% TSPP, Fisher Scientific, USA), sodium chloride (NaCl, Merck, Vienna, Austria), nitric acid (HNO_3_, 70%, Merck, Austria), sodium hydroxide (NaOH, Merck, Austria).

**Table 1:** List of rock and mineral standards used in this study and their relevant mineral types, sources and publications of certification or analysis

Standard material	Sample type	Source/reference
IAEA-B-8	Natural clay	Internation Atomic Energy Agency, Austria
JA-2	Andesite	Geological Survey of Japan^24^
JB-2	Basalt	Geological Survey of Japan^24^
IMt-2	Illite	Clay Minerals Society, Source Clays Repository^25^
MESS-4	Marine sediment	National Research Council, Canada^26^
RS3-BAM	Calcite (CaCO_3_)	Federal Institute for Materials Research and Testing (BAM), Germany

### Preparation of rock and mineral standards

0.5 g of several powdered mineral standard materials (IAEA-B-8, JA-2, JB-2, IMt-2, MESS-4, RS3-BAM, Table 1) were each accurately weighed and immersed in 10 mL of ultrapure water. The samples were sonicated for 30 seconds to suspend small particles shortly before analysis. To each suspension, an aliquot of 100 nm Au NPs was spiked (25 μL into 10 mL) and the resulting suspensions were diluted by a factor of between 40–4 000 (Au particles) and 5 000–20 000 (Fe-containing particles) times depending on the analyte concentration in the sample. These dilutions were determined by performing serial dilutions of samples until particles were being detected above blank levels. A conservative maximum detection rate of 1000 particles per min^−1^ was set to reduce the likelihood of double events (2 particles being detected as 1) occurring. Following spiking, the suspensions were split into three subsamples which were then either filtered by syringe (0.22 μm, nylon, Watman, GE healthcare Life Sciences, UK), centrifugated (1000*g*) or left untreated. An ultrapure water control was employed and was treated in the same manner (*i.e.*, spiked with Au NPs, split into three groups of untreated, filtered, centrifugated). The samples were then investigated by SP ICP-TOFMS.

### Instrumentation and software

Non-target particle screening was conducted using a Vitesse-series ICP-TOFMS instrument, equipped with a concentric nebuliser and cyclonic spray chamber (Nu Instruments, Wrexham, UK),was used to analyse particles in the mineral extract. The analysis was conducted at the ice core LA-ICP-MS facility of the Alfred-Wegener Institute (Bremerhaven, Germany). Each sample was measured once for 1 minute each with an integration time for all analyses of 0.1 ms, four spectra were binned before saving data to disk. A mass range from 28 to 220 *m*/*z* was monitored. ICP-TOFMS instrumental parameters were RF power = 1350 W, coolant gas flow = 13 L min^−1^, auxiliary gas flow = 2 L min^−1^, nebuliser gas flow = 1.15 L min^−1^, collision/reaction cell (CRC) RF set = 2.0 V, CRC gas flows: He = 14 L min^−1^, H_2_ = 10 L min^−1^.

Following non-target particle screening a targeted approach was employed. Relative particle recoveries of selected particulate elements (Au and Fe) were investigated by SP ICP-MS/MS using an Agilent 8900 ICP-MS/MS (Agilent Technologies, USA) at the University of Graz. The analysis of ^56^Fe was conducted with the addition of H_2_ (2 mL min^−1^); no gas was added for ^197^Au analysis. ICP-MS/MS parameters: RF Power = 1550 W, nebuliser gas flow 0.95 L min^−1^, integration time = 0.1 ms.

Data analysis was carried out using SPCal (V1.2.2); a python-based software designed to process single-event ICP-quadrupole-MS (ICP-QMS) and ICP-TOFMS data.^10,27^ A detection threshold (*α* = 10^−6^) was used, and Poisson and compound Poisson statistics were used for SP ICP-MS/MS and SP ICP-TOFMS, respectively. For the latter, compound Poisson distributions were modelled using a lognormal approximation as presented by Lockwood *et al.*^10^ More detailed information is available within the literature^10,28^ For Au nanoparticles, the size detection limit (LD_size_) was stable across all analysis days and ranged from 15–20 nm. Fe particle analysis was influenced more often by ionic background differences between the matrices. The calculated limits of detection are stated for each analysis in the supplementary (Tables S1–S3). With decision limits calculated it was then possible to assign any distinct signal which occurs above the limit as a single particle.

### Non-target particle screening (SP ICP-TOFMS)

A non-target NP screening approach was taken as described previously in the literature.^8,10^ Briefly, it is possible within SPCal to set and adjust a non-target screening filter which can be used to flag any element which is detected a given number of times. The screening parameters selected for this purpose were 100 ppm for 1 million events, *i.e.*, for the first 1 million data points if an element shows signals above the critical limit in 100 instances it will be flagged by the software.^10^ In this case several elements were identified across the sample series (Table 1), these included Fe, Si, Al, Ti, Ba, Zr, Ce, Mg and the spiked Au particles; Fe, Al, Si and Au were qualitatively identified as those present in the highest concentrations.

Mixed ionic standard solutions calibration (0–80 μg L^−1^, 5 data points) and an ultra-pure water blank were analysed to determine ionic responses (counts per μg L^−1^) for any detected element. A calibration standard was reanalysed after every 20 samples to monitor instrumental drift with no significant drift in ionic response being detected across a single day of analysis. This was prepared using a multi-element standard (ICP multi-element standard solution IV, Merck, Austria); a separate ionic solution was prepared for Au to avoid precipitation. A gravimetric approach was used to determine the sample uptake rate (0.442 mL min^−1^). A 2% transport efficiency was determined, *via* the “size method”,^29^ whilst monitoring a 100 nm Au NP suspension in ultrapure water.

### Target analysis of naturally formed particles by SP ICP-MS

Various elements were identified to be present as particles using the non-target screening approach and included for example Al, Ti, Si and Fe were all present in high concentrations. Fe was detected in all samples and was chosen for more in-depth analysis as it was present across a large number concentration range. Furthermore, it has an immense importance in various geochemical and biological cycles as well as several mineral phases which respond differently to the chosen experimental conditions. As such, targeting Fe, we expected to see discriminating effects for different minerals and thus demonstrate how different preparation techniques induce artefacts and how a critical particulate element responds to simple variations in sample preparation strategies.

Thus, Fe-containing particles as a common natural mineral entity and Au as a typical model particle were chosen as target analytes. A range of common or practical chemical treatments were tested to promote particle stability during filtration or centrifugation. This included surfactants and extractant solutions which were previously applied in the literature to enhance particle stability from improved electrostatic or steric interactions which can help to prevent particle agglomeration. Seven specific conditions/additives which were investigated to suspend and stabilise particles, these included: Triton X-100 (1.5% m v^−1^),^30–32^ sodium dodecyl sulphate (SDS, 0.05%, m v^−1^)^33,34^ and tetrasodium pyrophosphate (TSPP, 2.5 mmol).^20^ Additionally, pH adjustments (pH 4 and pH 9) and increased salinity (NaCl, 0.01 mmol) were chosen as practical strategies to investigate the impact of different ionic strength and surface potentials within the systems. Adjustments of pH were made through the addition of HNO_3_ and NaOH solutions. Particle recoveries were determined by taking the ratio of the treated sample (*i.e.*, a sample which was extracted, filtered, diluted and measured) to the untreated sample (recovery% = PNC_treated_/PNC_untreated_ × 100). The original list of standard materials (Table 1) and 1 MilliQ control were then each split into 7 groups, each one being treated with one of the chemical procedures listed above, resulting in 42 different sample types (each of which had been spiked with 100 nm Au NPs) which were then again split and either left untreated, syringe filtered or centrifugated. Fig. S1 displays this information graphically as a flow diagram.

Following the identification of Fe and Au as target elements, a SP ICP-MS/MS method was used additionally to take advantage of the improved ion transmission relative to the TOF. For SP ICP-MS/MS, analysis transport efficiencies were again calculated as described above for SP ICP-TOFMS. These analyses took place over five days with the transport efficiency being determined daily. Transport efficiencies ranged from 2–9% across the five days of analysis.

For Au analysis, there were no detectable particles in the blanks; however, for Fe in some cases particles were detectable in the measured blank solutions. To account for this, eqn (1) was adapted from Laborda *et al.*,^35^ where *σ*_N,B_ is the standard deviation of the blank sample, *η* is the transport efficiency (2–9%), *Q*_sam_ is the uptake rate (0.44 mL min^−1^) and *t*_i_ is the acquisition time (1 minute). This resulted in a LD_PNC_ of between 1 × 10^5^ and 5 × 10^5^ particles per L^−1^.1
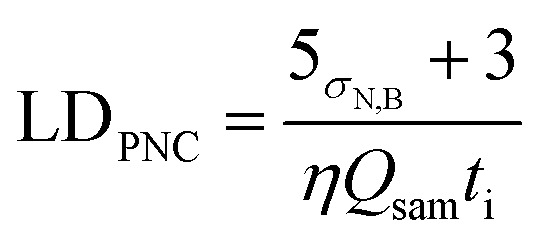


## Results and discussion

### Particle recovery by SP ICP-TOFMS

As the aim of this study was to probe sample preparation methods for particle analysis in environmentally relevant samples, it was necessary to use samples, which could mimic a sample collected from the field. A series of rock and mineral standards were selected, extracted in ultra-pure water and spiked with 100 nm Au standards. Additionally, a control was prepared by spiking 100 nm Au NPs into ultra-pure water. This approach generated samples, which met three key criteria: they contained a complex matrix (rock powder extracts), well-characterised particles (spiked Au), and naturally formed particles. Having these three components enabled the evaluation of particle recoveries and the comparison between synthesised and naturally occurring particles all within a matrix which is somewhat similar to an environmental sample collected in the field. Samples were then divided into three subsamples: untreated, 0.22 μm filtered and centrifugated and finally analysed by SP ICP-TOFMS.

The first step in the analysis of the selected sample was a scoping experiment, which aimed to identify analytes of interest. In recent years, non-target screening methods have been developed specifically for single event ICP-MS analysis.^8,10^ Applying a non-target screening approach to the water extracts of a series of selected standard materials,^8,10^ identified a range of potential NP in the untreated samples. These included naturally occurring particles of Fe, Al, Si, Ti, Ba, Ce, Mg and the spiked Au particles. However, following both centrifugation and syringe filtration, it was immediately apparent that the non-target screening was able to detect fewer elements, ergo particle species were being lost through sample preparation. This is visible in Fig. S2, which shows the non-target screening user interface of SPCal,^10^ highlighting elements which are present above the set threshold (100 ppm, minimum number of events = 1 million). In this example, following centrifugation it was apparent that Mg, Ba and even the Au spike were no longer flagged by the software. In order to quantify recovery, it was necessary to identify an element that was sufficiently abundant to allow comparisons across all samples and conditions. Here, Fe was identified and was interrogated across the different extractions and conditions to underpin the requirement to consider artefacts and discriminating effects. Furthermore, Fe exists in a range of stable chemical forms in the samples (*e.g.*, Fe-oxides, Fe-rich silicates, Fe-sulphides) which may respond differently to chemical extraction or filtration. For example, hematite and pyrite are both common iron minerals but chemically different as one is an oxide and the other a sulphide. In some samples it was found that extractable particulate Fe was a major component (*i.e.*, MESS-4, marine sediment) and in others it was present in particles as a trace impurity (RS3-BAM) which was advantageous as it is more reflective of results from environmental sampling.

**Fig. 1:**
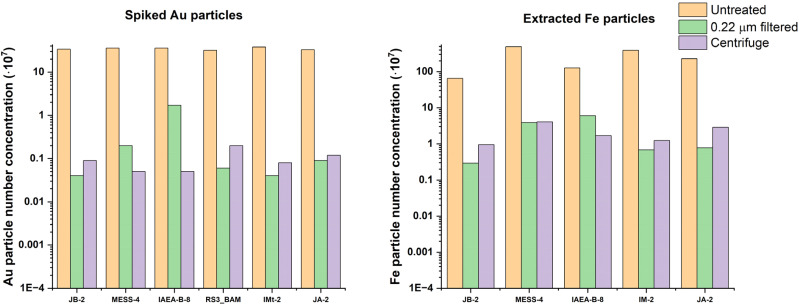
Particle number concentrations (particles per L^−1^) of spiked Au (left) and extracted Fe particles (right) found in water-extracted standards, comparing samples which were filtered (green), centrifugated (purple) and those which had no sample preparation beyond dilution in ultrapure water (orange).

The results of the analysis for Fe and Au relative particle recoveries following centrifugation and syringe filtration are displayed in Fig. 1. It is apparent that in all cases, filtration (green) and centrifugation (purple) resulted in the loss of most of the detectable particles, relative to the untreated samples (orange). In a majority of the samples shown in Fig. 1, PNC dropped by over 99% for both preparation strategies despite a nominal cut-off (220 nm) well above the expected particle size (Au, 100 nm).

This effect of losing most of the particulate analytes through sample preparation has many implications which are detrimental for the analysis of environmental samples, the most obvious of which is accurate particle number calibration and the characterisation of mass and size distributions. Evaluating particle concentrations by SP ICP-MS already has its challenges, particularly with estimating crucial details such as particle transport efficiencies and detection thresholds; however, improper sample preparation may have an even larger impact on data quality. A second layer to the issue of losing a substantial number of particles through filtering or centrifugation is the impact of dilution factors. Environmental samples are often soil extracts or brine solutions which must be diluted to deal with the complex matrix, and which contain both the particulate matter of interest and high concentrations of ionic species. If 90% of the total particle count has been lost from preparative steps, then it is inevitable that the dilution factor will have to be lower which will impact detection limits through the increase in ionic backgrounds.

**Fig. 2:**
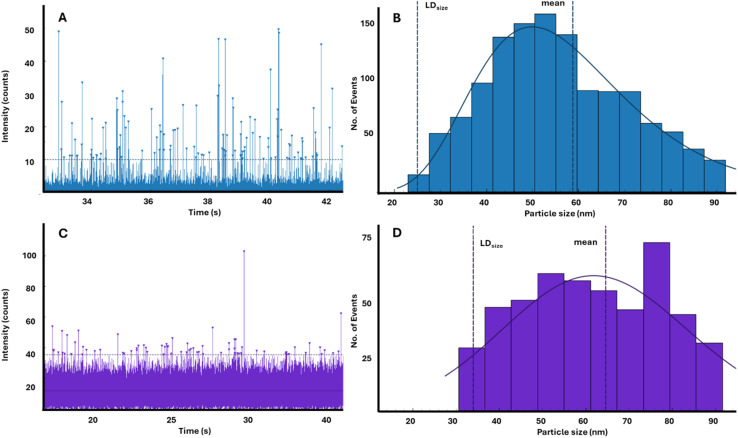
Practical impact on particle detection following the use of syringe filtration *versus* an untreated sample for naturally formed particles. (A) Raw data for Fe from the direct analysis of the rock sample, MESS-4 (marine sediment). (B) Shows the particle size histogram for the raw data presented in 2A, here the particles were assumed to be spheres with the density of Fe_2_O_3_ (5.24 g cm^−3^) to generate the histogram with a limit of size detection (LD_size_ = 24 nm) (C) Raw data for the same sample, following filtration and lower dilution (*x*50 less) (D) Size histogram for the particles shown in 2C, a visual shift in the histogram occurs due to the spike in ionic background (LD_size_ = 34 nm).

To highlight this issue, results from the analysis of the marine sediment sample, MESS-4, are shown in Fig. 2. Initial values for the Fe ionic background for MESS-4 were 100 ± 40 ng Fe L^−1^; however, syringe filtration resulted in a drop in total particle numbers and, therefore, lower dilution factors were necessary to still detect Fe particles (50× less diluted). This process led to ionic background values increasing to 3900 ± 9000 ng Fe L^−1^. As a result, the limit of size detection (LD_size_) rose from 25 nm to 34 nm, assuming a Fe_2_O_3_ phase. These effects are visualised in Fig. 2. In 2A and 2B raw data and subsequently generated size distribution for Fe particles from a water extract of the MESS-4 standard are presented. Fig. 2C and D show the results from filtering the same sample prior to analysis. The increase in ionic background is immediately apparent when comparing 2A and 2C with many of the smallest particles which are visible in 2A disappearing in the higher noise of 2C. Furthermore, 2D displays a shift in the size distribution as the smallest particles are lost to the increased background.

This data indicates that the commonly applied preparative techniques will lead to the loss of a majority of all particles; however, these techniques will likely continue to be applied due to the complexity of many environmental matrices. Therefore, strategies designed to enhance particle stability and recovery throughout sample preparation are required.

### Impact of additives on extracted particles

Particle properties and stabilities were significantly impacted by matrix and spiked additives, which was evaluated in various studies beforehand.^36,37^ One aim was to understand the importance of electrostatic interactions, which are influenced by the zeta potential, and to prevent unwanted events such as agglomeration.^38,39^ One common approach in the analytical toolkit for increasing particle stability is the manipulation of particle surface charges and interactions by introduction of a surfactant like SDS or Triton X-100 as well as by changing salinity and pH. Following experiments to study particle filtration, we investigated whether the addition of additives could improve stability and as such recovery. It is known that the stabilisation through surfactants is much dependent on the particle species and so far, mostly synthetic model particles have been subjected to more rigorous tests.^31,34,39^ The analysis of suspended mineral/sediment materials enabled us to consider particles which are likely present in the environment. We investigated seven treatment options: untreated, Triton x100, SDS, TSPP, NaCl, pH 4, and pH 9. Each method was chosen to explore simple strategies for enhancing system stability and promote particle suspension through the improvement of electrostatic interactions. The Zeta potential is a fundamental characteristic of a particle which is affected by the nature of the particle (*e.g.*, mineralogy) but also the pH and ion strength of the matrix. Given that around point of zero charge (*i.e.*, neutral particle) particles may agglomerate, changing the pH a positive (low pH) or negative (high pH) charge may improve overall stability through electrostatic repulsion.^40^ An ultra-pure water (MilliQ) sample spiked with Au NPs was used as a control. Samples were split into the same subcategories: untreated, 0.22 μm filtered and centrifugated.

**Fig. 3:**
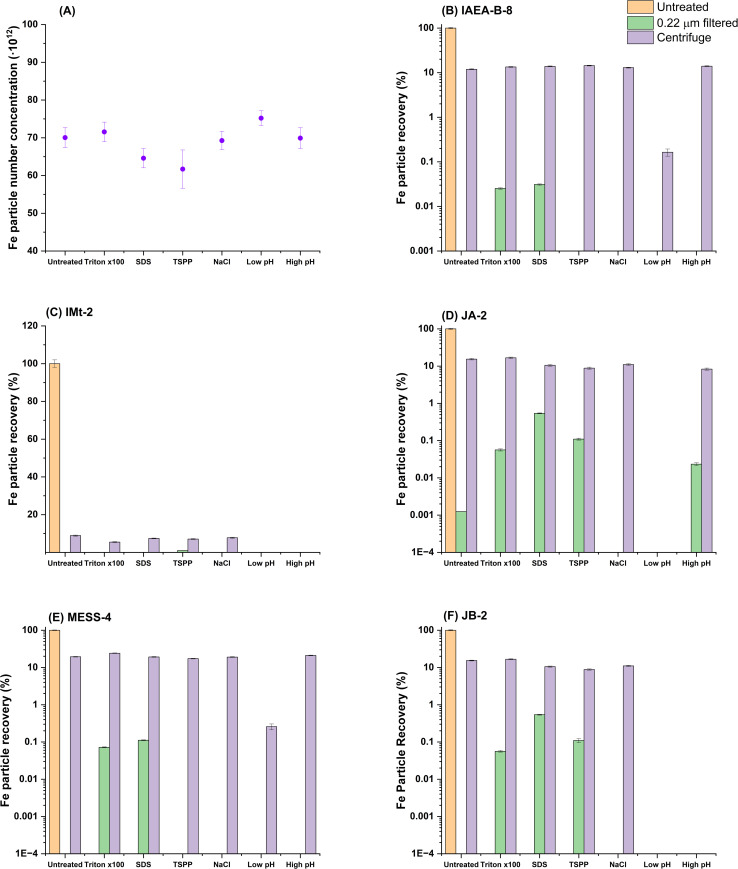
(A) Plot of Fe PNC in the sample IAEA-B-8 showing stability in the detectability of Fe when using various chemical treatments with no sample preparation (presented as particles). (B–F) Fe particle recoveries from the listed standards (B) IAEA-B-8 (C) IMt-2 (D) JA-2 (E) MESS-4 (F) JB-2. Particle recoveries were normalised to the direct analysis of the sample (*i.e.*, 3B = IAEA, water extract, diluted and measured) with no further sample preparation strategies employed. Recoveries were determined (as described in eqn (1) in the methods, by dividing particle numbers for the treated samples by the untreated (orange). All associated data to generate this figure is presented in Tables S1–3.

Fig. 3A uses the IAEA-B-8 reference material as an example of how the sample modifications impacted the estimated particle number concentrations for Fe-bearing particles when no further preparation strategies were employed (*i.e.*, analysis of IAEA-B-8 with various additives but no filtration or centrifugation). Interestingly, the determined Fe PNCs were relatively stable across each sample type regardless of sample preparation. As such, the results presented in Fig. 3B–F were normalised to the untreated sample, which had no sample preparation beyond dilution. This sample type represents the closest possible example to direct analysis and likely is most representative of the “true” concentration.

When evaluating spiked Au and extracted Fe from a variety of sample types, both filtration and centrifugation returned similar particle recoveries relative to the unfiltered sample. With most cases resulting in more than 99% of the particles being lost (compare Fig. 1). In Fig. 3, this trend was maintained for the Fe particles and there were no real improvements in terms of relative particle recovery for Fe particles from any of the applied treatments. Centrifugation showed inter-sample variation with particle recoveries of between 5 and 25% being found across all sample types, suggesting an effect of sample matrix or perhaps particle composition (*i.e.*, different Fe species) on the outcome of sample preparation. In some cases, pH adjustment proved to result in total loss of particles, presumably this was due to dissolution of Fe_2_O_3_ (s) to Fe^2+^ at low pH or the formation of aggregates. There was a bias towards the loss of larger particles with median particle size dropping in all cases following centrifugation by average of 13% (±7%) compared to the untreated samples. As such, it is to be expected that in polydisperse samples, centrifugation could result in a severe underestimation of particle size. These results are contrary to those in a study by Jreije *et al.*,^22^ who found recoveries of between 40 and 80% in river and rainwater for centrifugation at 1000*g*. This may be a result of differences in the particles with their strategy employing 10 nm particles while our study focused on natural, polydisperse, and larger particles; equally this could be a result of from investigating different chemical species. Nevertheless, even in the instance of the Jreije *et al.* study it is apparent that in the “best case” scenario for particle analysis in an environmental context, consistently achieving quantitative recoveries near 100% is not probable.

### Impact of sample preparation on reference particles

Au NPs represent a different aspect to this investigation; Au NPs are among the most characterised nanomaterials and are very often used for calibration or as internal standards. In this study, they were used in an internal standard fashion and spiked to study recovery and fractionation differences for an engineered particle species.

**Fig. 4:**
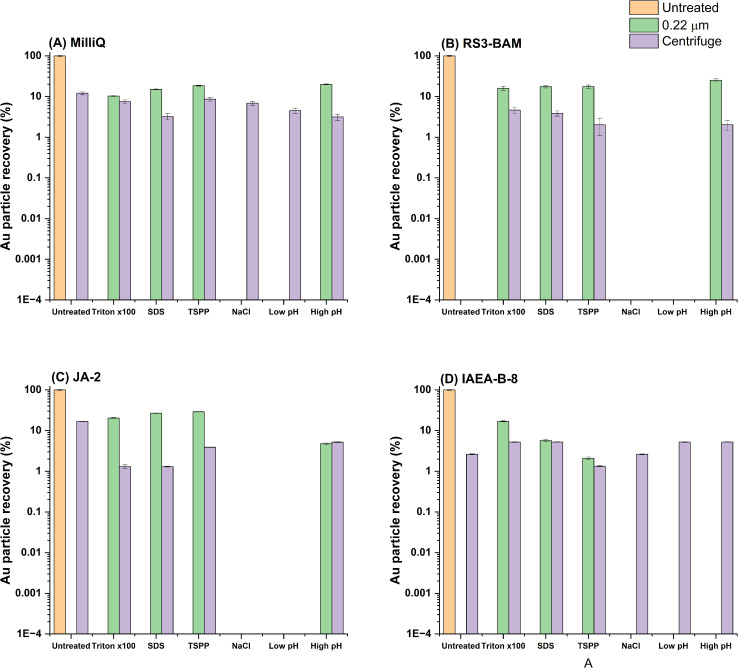
Particle recoveries for spiked 100 nm Au NPs in four different matrices: (A) MilliQ, (B) RS3-BAM, (C) JA-2, (D) IAEA. The figure is normalised to the direct analysis of the sample (*i.e.*, 4A = IAEA, water extract, spiked with Au, diluted and measured) with no further sample preparation strategies employed. Relative particle recoveries are expressed on a log scale. All associated data to generate this figure is presented in Tables S4–6.

Fig. 4 highlights a key difference between the Au NPs and the extracted Fe particles. Like Fe, when left untreated, Au NPs were entirely removed from the matrix by filtration. Any attempted addition of NaCl, or pH adjustment was unsuccessful in almost all cases. However, when SDS, TSPP or Triton X-100 was added to the matrix particle recoveries increased from <1% to up to 29% in some cases. Average particle size across all investigated matrices for the Au particles which were stabilised through the addition of SDS, TSPP or Triton X-100 was 89 nm ± 3 nm. As such there was an apparent underestimation of the particle size following the filtration of the particles.

Au particles responded similarly to the surfactant or pH treatment in each sample following filtration. Comparing Fig. 4A–D indicates that when Triton X-100 or SDS was added, a similar Au particle recovery was achieved. However, a greater degree of variability was visible in Fig. 3. In some cases, *i.e.*, 3D, TSPP was unsuccessful in stabilising the Fe particles and no significant recovery was achieved. Whereas 3E shows that TSPP resulted 1% of the Fe particles being recovered, relative to the untreated sample. This could be an effect of increased complexity in the Fe speciation in this sample. Nevertheless, this point emphasises the challenge when it comes to find a universial approach to recover Fe without *a priori* knowledge (*e.g.*, in environmental scenarios).

The behaviour of Au particles during centrifugation (Fig. 4, purple) was comparable to the that of Fe particles as indicated in Fig. 3. Although, the average particle recovery was found to be 3% (±3%), which is lower than the average particle recovery described for Fe at (Welch's *t*-test *p* < 0.001). This difference can likely be attributed to the difference in densities. Here, it is assumed that the Fe particles were present as Fe_2_O_3,_ which was the mineral phase according to the certification of the standards. The density of Fe_2_O_3_ (5.24 g cm^−3^) is 3.6 times less than that of the Au reference particles (19.3 g cm^−3^), as such it is expected that even under the gentle conditions which were applied (1000 g) the denser particles would respond less favourably to centrifugation.

Au and Fe particles responded differently to both filtration and centrifugation strategies. Filtration impacted Fe particle recoveries significantly more resulting in over 99% of the particles being lost in most cases, while centrifugation was found to result in fewer Au particles being recovered. Further differences were found from the chemical treatment approaches with Au NPs showing superior stability when filtered in the presence of TSPP, SDS or Triton X-100. This suggests that broad, simple approaches to environmental particle analysis is difficult and using well defined particles as an internal standard may not be reflective of the extracted particles in all cases.

### Implications on environmental analysis and recommendations

Considering the data in this study alongside those in the literature, which have considered sample preparation in some detail,^22,23^ shines a light on a clear problem within the single particle community; sample preparation strategies often result in a significant particle losses and species-specific effects may need to be taken into account. Previous studies were able to achieve particle recoveries of over 50% in some, but not all, cases. However, to achieve this it was a prerequisite to understand the particle compositions and for those particles to have favourable properties (*i.e.*, size, stability). No previous attempt had looked at naturally formed particles and it is evident from the data we have presented here that naturally formed particles (in this case Fe-bearing particles) do not necessarily respond the same to sample preparation strategies as particle standards (Au). In fact, where the relative recoveries of the particle standard (Au nanoparticles) were constant across each sample type, Fe particles exhibited a greater variation. This variation highlights the increased complexity of naturally formed particles in environmental media, as in each case, analytes may be present in a range of species with different characteristics (*i.e.*, zeta potential, density, shape and size). The findings contained within this study are of critical importance and it is clear that environmentally focused particle research must consider the impacts of standard sample preparation approaches. Failing to take preparation artefacts into account may lead to inevitable and irreversible alterations made to the sample. Sample preparation strategies can clearly result in the underestimation of particle number concentrations and distortion of particle size determinations makes quantitative analysis by SP ICP-MS in an environmental context a serious challenge which has not yet been solved. Quantitative analysis is made more of a challenge by the absence of environmentally relevant reference nanomaterials which are certified in terms of both particle number and size.

To date, the only approach which could result in representing an environmental sample effectively is to avoid sample preparation except for dilution, which can also lead to the loss of analyte with changes in ionic strength leading to a drop in particle stability.^9^ Even strategies which use matrix matching and external particle calibration are limited due to differences between natural and synthesised particles. It may be feasible to perform a non-target screening of a sample, generate a picture of analytes of interest and then modify sample preparation from there to optimise for a given target. Otherwise, there are analytical strategies which could circumvent these problems, such as the application of laser ablation-SP ICP-MS^41^ or microextraction^42^ which are capable of detecting particles directly from a filter surface; strategies such as these may be capable of turning the downsides of filtration into a viable strategy for investigating naturally occurring particles in the environment. Although, as with each analytical technique, these approaches also have limitations and inherent challenges.

## Conclusions

The aims of this study were to investigate how sample preparations strategies impact both naturally occurring and well-characterised model NPs. To do so, water extracts were performed on a series of materials which were found to contain Fe nanoparticles across a broad concentration range by SP ICP-TOFMS and SP ICP-MS/MS were spiked with 100 nm Au NPs. These extracts were then exposed to a range of sample preparation strategies and additives including the addition of surfactants, pH adjustment, syringe filtration and centrifugation. As a result, it was determined that in all cases where filtration or centrifugation was employed a vast majority of particles were lost. For Au NPs, the addition of TSPP, SDS or Triton X-100 was effective in increasing particle number recoveries to up to 28%. For naturally occurring particles, SP ICP-TOFMS successfully showed that following filtration particles which are present in the lowest concentrations (*i.e.*, Ti, Ba, Ce) in a sample become undetectable following centrifugation or filtration. Therefore, when applying these preparative strategies, it would be impossible to characterise particles present in low abundances in samples, analysis of which could be critical. The targeted analysis of Fe by SP ICP-MS/MS found that filtration reliably resulted in the loss of >99% of the particles, highlighting that it is likely that comparing naturally formed nanoparticles to model nanoparticles with well-known properties is not guaranteed to be effective. This suggests that the selection or use of particulate internal standards to monitor sample preparation and analysis may be an ineffective practice. It is evident that for studies where the quantification of particle number concentrations in environmental samples is a goal, it is favourable to avoid simple, traditional sample preparation strategies.

## Conflicts of interest

There are no conflicts of interest to declare.

## Supplementary Material

JA-040-D5JA00170F-s001

## Data Availability

The raw data used for this manuscript has been made available at https://osf.io/5rkzj/files/osfstorage/6810333a2411394daa3cbf13 as a compressed folder. The total size of the data is approximately 100 GB. The software used to process this data is open access and available at https://github.com/djdt/spcal/releases/. The data is labelled according to the sample preparation treatment and typically the dilution factor applied. The nature of the data could make it difficult to evaluate without guidance but it is possible. The SI contains supporting figures and tabulated data which supports figures generated for use in the main text. See DOI: https://doi.org/10.1039/d5ja00170f.

## References

[cit1] Bundschuh M., Filser J., Lüderwald S., McKee M. S., Metreveli G., Schaumann G. E., Schulz R., Wagner S. (2018). Nanoparticles in the environment: where do we come from, where do we go to?. Environ. Sci. Eur..

[cit2] Andrady A. L. (2011). Microplastics in the marine environment. Mar. Pollut. Bull..

[cit3] Hassellöv M., Readman J. W., Ranville J. F., Tiede K. (2008). Nanoparticle analysis and characterization methodologies in environmental risk assessment of engineered nanoparticles. Ecotoxicology.

[cit4] Filipe V., Hawe A., Jiskoot W. (2010). Critical Evaluation of Nanoparticle Tracking Analysis (NTA) by NanoSight for the Measurement of Nanoparticles and Protein Aggregates. Pharm. Res..

[cit5] Montaño M. D., Olesik J. W., Barber A. G., Challis K., Ranville J. F. (2016). Single Particle ICP-MS: Advances toward routine analysis of nanomaterials. Anal. Bioanal. Chem..

[cit6] Mozhayeva D., Engelhard C. (2020). A critical review of single particle inductively coupled plasma mass spectrometry-A step towards an ideal method for nanomaterial characterization. J. Anal. At. Spectrom..

[cit7] Lee S., Bi X., Reed R. B., Ranville J. F., Herckes P., Westerhoff P. (2014). Nanoparticle size detection limits by single particle ICP-MS for 40 elements. Environ. Sci. Technol..

[cit8] Gonzalez de Vega R., Lockwood T. E., Paton L., Schlatt L., Clases D. (2023). Non-target analysis and characterisation of nanoparticles in spirits *via* single particle ICP-TOF-MS. J. Anal. At. Spectrom..

[cit9] Gonzalez de Vega R., Lockwood T. E., Xu X., Gonzalez de Vega C., Scholz J., Horstmann M. (2022). *et al.*, Analysis of Ti- and Pb-based particles in the aqueous environment of Melbourne (Australia) *via* single particle ICP-MS. Anal. Bioanal. Chem..

[cit10] Lockwood T. E., Schlatt L., Clases D. (2025). SPCal – an open source, easy-to-use processing platform for ICP-TOFMS-based single event data. J. Anal. At. Spectrom..

[cit11] Goodman A. J., Gundlach-Graham A., Bevers S. G., Ranville J. F. (2022). Characterization of nano-scale mineral dust aerosols in snow by single particle inductively coupled plasma mass spectrometry. Environ. Sci. Nano.

[cit12] Ramos K., Ramos L., Gómez-Gómez M. M. (2017). Simultaneous characterisation of silver nanoparticles and determination of dissolved silver in chicken meat subjected to *in vitro* human gastrointestinal digestion using single particle inductively coupled plasma mass spectrometry. Food Chem..

[cit13] Furtado L. M., Hoque M. E., Mitrano D. F., Ranville J. F., Cheever B., Frost P. C. (2014). *et al.*, The persistence and transformation of silver nanoparticles in littoral lake mesocosms monitored using various analytical techniques. Environ. Chem..

[cit14] Donovan A. R., Adams C. D., Ma Y., Stephan C., Eichholz T., Shi H. (2016). Single particle ICP-MS characterization of titanium dioxide, silver, and gold nanoparticles during drinking water treatment. Chemosphere.

[cit15] Echegoyen Y., Nerín C. (2013). Nanoparticle release from nano-silver antimicrobial food containers. Food Chem. Toxicol..

[cit16] Peters R. J. B., Van Bemmel G., Herrera-Rivera Z., Helsper H. P. F. G., Marvin H. J. P., Weigel S. (2014). *et al.*, Characterization of titanium dioxide nanoparticles in food
products: Analytical methods to define nanoparticles. J. Agric. Food Chem..

[cit17] Bai Q., Li Q., Liu J. (2023). Determination of the Particle Number Concentration, Size Distribution, and Species of Dominant Silver-Containing Nanoparticles in Soils by Single-Particle ICP-MS. Environ. Sci. Technol..

[cit18] Loeschner K., Navratilova J., Købler C., Mølhave K., Wagner S., Von Der Kammer F. (2013). *et al.*, Detection and characterization of silver nanoparticles in chicken meat by asymmetric flow field flow fractionation with detection by conventional or single particle ICP-MS. Anal. Bioanal. Chem..

[cit19] jie C. Y., Shih Y. hsin, Su C. H., Ho H. C. (2017). Comparison of three analytical methods to measure the size of silver nanoparticles in real environmental water and wastewater samples. J. Hazard. Mater..

[cit20] Zhou X., Xiao Q., Deng Y., Hou X., Fang L., Zhou Y., Fangbai L. (2024). Direct evidence for the occurrence of indigenous cadmium-based nanoparticles in paddy soils. Sci. Total Environ..

[cit21] Nowack B., Bucheli T. D. (2007). Occurrence, behavior and effects of nanoparticles in the environment. Environ. Pollut..

[cit22] Jreije I., Hadioui M., Wilkinson K. J. (2022). Sample preparation for the analysis of nanoparticles in natural waters by single particle ICP-MS. Talanta.

[cit23] Liu H., Jia R., Xin X., Wang M., Sun S., Zhang C. (2023). *et al.*, Single particle ICP-MS combined with filtration membrane for accurate determination of silver nanoparticles in the real aqueous environment. Anal. Sci..

[cit24] Imai N., Terashima S., Itoh S., Ando A. (1995). 1994 COMPILATION OF ANALYTICAL DATA FOR MINOR AND TRACE ELEMENTS IN SEVENTEEN GSJ GEOCHEMICAL REFERENCE SAMPLES, “IGNEOUS ROCK SERIES.”. Geostand. Newsl..

[cit25] Vogt C., Lauterjung J., Fischer R. X. (2002). Investigation of the Clay Fraction (<2 μm) of the Clay Minerals Society Reference Clays. Clays Clay Miner..

[cit26] Willie S. , Nadeau K., Pihillagawa Gedara I., Yang L., Clancy V., Grinberg P., Kumkrong P., Mercier P. H. J., Mihai O., Tyo D. D., Jiang C., Kingston D. M., Meija J., Maxwell P. and Mester Z., Certificate of Analysis Certified Reference Material MESS-4 Marine Sediment Certified Reference Material for total and extractable metal content MESS-4 is a marine sediment Certified Reference Material (CRM) from the National Research, 2014, 10.4224/crm.2014.mess-4

[cit27] Lockwood T. E., Gonzalez De Vega R., Clases D. (2021). An interactive Python-based data processing platform for single particle and single cell ICP-MS. J. Anal. At. Spectrom..

[cit28] Gundlach-Graham A., Lancaster R. (2023). Mass-Dependent Critical Value Expressions for Particle Finding in Single-Particle ICP-TOFMS. Anal. Chem..

[cit29] Pace H. E., Rogers N. J., Jarolimek C., Coleman V. A., Higgins C. P., Ranville J. F. (2011). Determining transport efficiency for the purpose of counting and sizing nanoparticles *via* single particle inductively coupled plasma mass spectrometry. Anal. Chem..

[cit30] Gaikwad D. S., Undale K. A., Patil D. B., Pore D. M., Kamble A. A. (2018). Triton X-100 stabilized Pd nanoparticles and their catalytic application in one-pot sequential Heck and Hiyama coupling in water. Res. Chem. Intermed..

[cit31] Santra S., Tapec R., Theodoropoulou N., Dobson J., Hebard A., Tan W. (2001). Synthesis and characterization of silica-coated iron oxide nanoparticles in microemulsion: The effect of nonionic surfactants. Langmuir.

[cit32] Dumbrava A., Berger D., Prodan G., Matei C., Moscalu F., Diacon A. (2017). The influence of Triton X-100 surfactant on the morphology and properties of zinc sulfide nanoparticles for applications in azo dyes degradation. Mater. Chem. Phys..

[cit33] Che M. H. S. N., Amir Z., Jan B. M., Khalil M., Azizi A. (2022). Colloidal Stability of CA, SDS and PVA Coated Iron Oxide Nanoparticles (IONPs): Effect of Molar Ratio and Salinity. Polymers.

[cit34] Vatanparast H., Samiee A., Bahramian A., Javadi A. (2017). Surface behavior of hydrophilic silica nanoparticle-SDS surfactant solutions: I. Effect of nanoparticle concentration on foamability and foam stability. Colloids Surf., A.

[cit35] Laborda F., Gimenez-Ingalaturre A. C., Bolea E., Castillo J. R. (2020). About detectability and limits of detection in single particle inductively coupled plasma mass spectrometry. Spectrochim. Acta, Part B.

[cit36] Nabweteme R., Yoo M., Kwon H. S., Kim Y. J., Hwang G., Lee C. H. (2015). *et al.*, Application of the extended DLVO approach to mechanistically study the algal flocculation. J. Ind. Eng. Chem..

[cit37] Rosenholm J. B. (2018). Evaluation of particle charging in non-aqueous suspensions. Adv. Colloid Interface Sci..

[cit38] Cao M., Liu Q., Chen M., Yang P., Xu Y., Wu H. (2017). *et al.*, Dispersing hydrophilic nanoparticles in nonaqueous solvents with superior long-term stability. RSC Adv..

[cit39] Kokot G., Bespalova M. I., Krishnan M. (2016). Measured electrical charge of SiO_2_ in polar and nonpolar media. J. Chem. Phys..

[cit40] Veloso C. H., Filippov L. O., Filippova I. V., Ouvrard S., Araujo A. C. (2020). Adsorption of polymers onto iron oxides: Equilibrium isotherms. J. Mater. Res. Technol..

[cit41] Holbrook T. R., Gallot-Duval D., Reemtsma T., Wagner S. (2021). An investigation into LA-spICP-ToF-MS uses for: *In situ* measurement of environmental multi-elemental nanoparticles. J. Anal. At. Spectrom..

[cit42] Bradley V. C., Burleson J., Andrews H. B., Thompson C. V., Spano T. L., Dunlap D. R. (2024). *et al.*, Mapping of uranium particles on J-type swipes with microextraction-ICP-MS. Analyst.

