# Supplementary material for: Assessing the impact of common sample preparation strategies for single particle ICP-MS regarding recovery and size distribution of natural single particles
Source: J Anal At Spectrom. 2025 Aug 20;40(10):2897–908. doi: 10.1039/d5ja00170f (PMC12426992; doi:10.1039/d5ja00170f)
Supplement: JA-040-D5JA00170F-s001 [file JA-040-D5JA00170F-s001.pdf]

Supplementary Information (SI) for Journal of Analytical Atomic Spectrometry.
This journal is © The Royal Society of Chemistry 2025

Supplementary Information

Assessing the impact of common sample preparation strategies for single particle ICP-MS regarding recovery and size distribution of natural single particles

Lhiam Paton[1]*, Sandra Kiesel[1], Grit Steinhoefel[2], Matthias Elinkmann[4], Thebny Thaise Moro[4], Raquel Gonzalez de Vega[1], Pascal Bohleber[2,3], David Clases[4]

[1] Trace Element Speciation Laboratory (TESLA), Institute for Analytical Chemistry, University of Graz, 8010 Graz, Austria.

[2] Alfred Wegener Institute Helmholtz Centre for Polar and Marine Research, 27570 Bremerhaven, Germany

[3] Department of Geosciences, Goethe University Frankfurt am Main, 60438 Frankfurt am Main, Germany

[4] nµLab, Institute for Analytical Chemistry, University of Graz, 8010 Graz, Austria

*E-mail: lhiam.paton@uni-graz.at

Table of Contents

Table S1. Particle number concentrations, median mass and median size for extracted Fe NPs in **untreated**. Values in brackets represent the limit of detection for the respective measurement. Each PNC and SD represent data from one minute of data collection.

Table S2. Particle number concentrations, particle recoveries, median mass and median size for extracted Fe NPs in **0.22 µm filtered** samples. Values in brackets represent the limit of detection for the respective measurement. Each PNC and SD represent data from one minute of data collection. Recoveries are calculated relative to the equivalent untreated sample in **Table S1**.

Table S3. Particle number concentrations, particle recoveries, median mass and median size for extracted Fe NPs in **centrifugated** samples. Values in brackets represent the limit of detection for the respective measurement. Each PNC and SD represent data from one minute of data collection. Recoveries are calculated relative to the equivalent untreated sample in **Table S1**.

Table S4. Particle number concentrations, and median size for Au spikes in **untreated** samples. Values in brackets represent the limit of detection for the respective measurement. Each PNC and SD represent data from one minute of data collection.

Table S5. Particle number concentrations, recoveries and median size for Au spikes in **0.22 µm filtered** samples. Values in brackets represent the limit of detection for the respective measurement. Each PNC and SD represent data from one minute of data collection.

Table S6. Particle number concentrations, recoveries and median size for Au spikes in **centrifugated** samples. Values in brackets represent the limit of detection for the respective measurement. Each PNC and SD represent data from one minute of data collection.

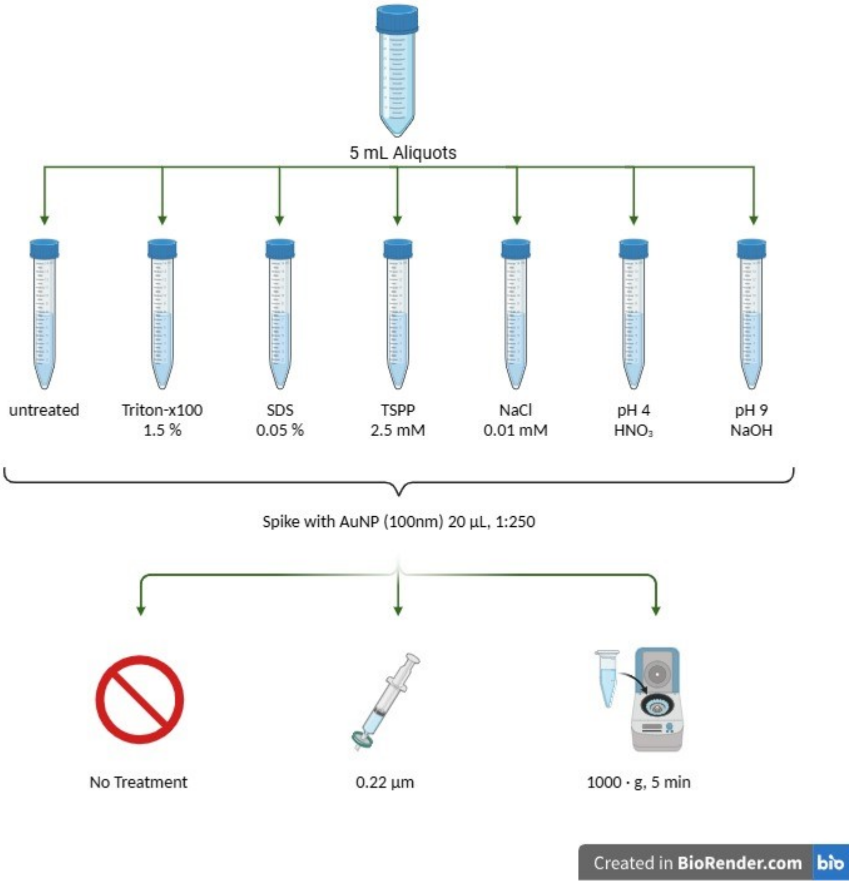

Figure S1. Flow diagram illustrating the sample preparation used for targeted SP ICP-MS analysis for extracted Fe and spiked Au particles, where 5 mL aliquots of rock standards extracted in water are treated with 7 sample preparations strategies before being spiked with Au NPs. The spiked solutions were then treated separately in each of the three ways shown.

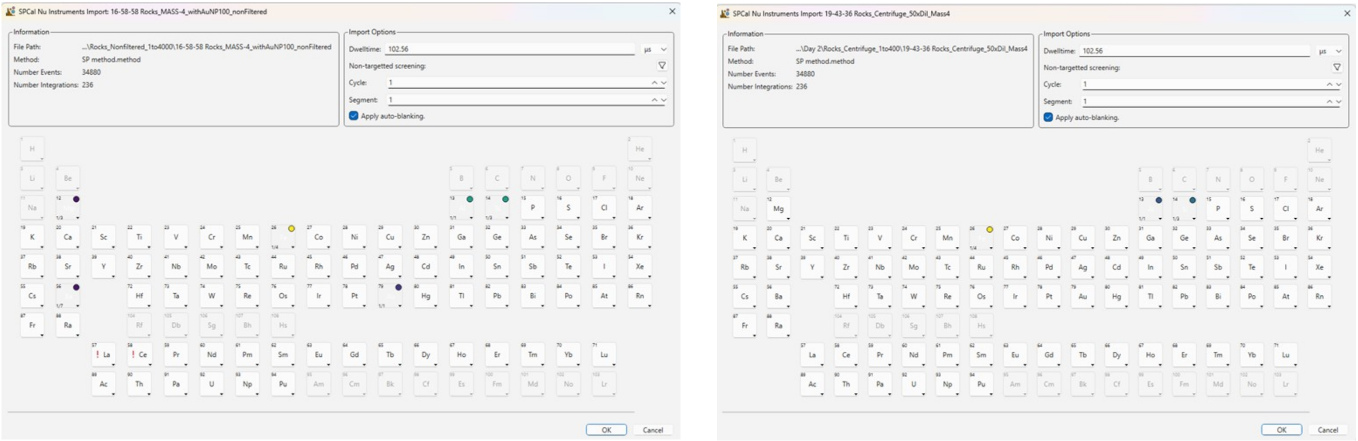

Figure S2. Left) non-target screening user interface showing the detection of Mg, Al, Si, Fe, Ba and Au particle events from the direct analysis of a marine sediment reference material (MESS-4). **Right)** equivalent user interface for the same sample (MESS-4) following gentle centrifugation (1000 · g), with half of the analytes now being undetectable. The centrifugated sample was 10x more concentrated relative to the untreated sample shown on the left.

Table S1. Particle number concentrations, median mass and median size for extracted Fe NPs in **untreated**. Values in brackets represent the limit of detection for the respective measurement. Each PNC and SD represent data from one minute of data collection. Where a "-" is present, there were not a sufficient number of events above the limit of detection.

	Treatment	PNC (particles·L^{-1}, ·10^7)	SD (·10^7)	Median Mass (fg)	Median Size (nm)
**IAEA-D-8**	**Untreated**	700000	9500	0.9 (LD = 0.1)	70 (LD = 36)
	**Triton X-100**	716000	9600	0.9 (LD = 0.1)	70 (LD = 36)
	**SDS**	646000	9100	0.8 (LD = 0.1)	70 (LD = 35)
	**TSPP**	617000	8900	0.9 (LD = 0.1)	70 (LD = 37)
	**NaCl**	693000	9400	0.9 (LD = 0.1)	70 (LD = 36)
	**pH 4**	752000	9900	0.9 (LD = 0.1)	70 (LD = 35)
	**pH 9**	699000	9500	0.9 (LD = 0.1)	70 (LD = 36)
**IMt-2**	**Untreated**	444000	7600	0.6 (LD = 0.1)	60 (LD = 35)
	**Triton X-100**	300000	6200	0.5 (LD = 0.1)	60 (LD = 35)
	**SDS**	325000	6500	0.5 (LD = 0.1)	60 (LD = 35)
	**TSPP**	217000	5300	0.5 (LD = 0.1)	60 (LD = 35)
	**NaCl**	370000	6900	0.6 (LD = 0.1)	60 (LD = 35)
	**pH 4**	328000	6500	0.5 (LD = 0.1)	60 (LD = 35)
	**pH 9**	372000	6900	0.5 (LD = 0.1)	60 (LD = 35)
**JA-2**	**Untreated**	96200	3500	0.7 (LD = 0.1)	60 (LD = 34)
	**Triton X-100**	107000	3700	0.7 (LD = 0.1)	70 (LD = 34)
	**SDS**	67400	2900	0.7 (LD = 0.1)	60 (LD = 34)
	**TSPP**	66400	2900	0.7 (LD = 0.1)	70 (LD = 34)
	**NaCl**	84400	3300	0.8 (LD = 0.1)	70 (LD = 34)
	**pH 4**	15800	1400	1.1 (LD = 0.1)	70 (LD = 34)
	**pH 9**	233000	5500	1.2 (LD = 0.1)	80 (LD = 35)
**JB-2**	**Untreated**	133000	3500	1.0 (LD = 0.1)	70 (LD = 34)
	**Triton X-100**	157000	3700	1.0 (LD = 0.1)	70 (LD = 35)
	**SDS**	149000	2900	1.0 (LD = 0.1)	70 (LD = 34)
	**TSPP**	142000	2900	0.8 (LD = 0.1)	70 (LD = 34)
	**NaCl**	118000	3300	0.9 (LD = 0.1)	70 (LD = 34)
	**pH 4**	66200	1400	1.0 (LD = 0.1)	70 (LD = 34)
	**pH 9**	72800	5500	1.2 (LD = 0.1)	80 (LD = 34)
**MESS-4**	**Untreated**	445000	7600	0.7 (LD = 0.1)	60 (LD = 35)
	**Triton X-100**	489000	7900	0.7 (LD = 0.1)	60 (LD = 36)
	**SDS**	424000	7400	0.7 (LD = 0.1)	60 (LD = 35)
	**TSPP**	346000	6700	0.6 (LD = 0.1)	60 (LD = 36)
	**NaCl**	480000	7900	0.7 (LD = 0.1)	60 (LD = 35)
	**pH 4**	572000	8600	0.8 (LD = 0.1)	60 (LD = 36)
	**pH 9**	472000	7800	0.7 (LD = 0.1)	60 (LD = 35)
**R6M-1**	**Untreated**	516	42	1.3 (LD = 0.1)	80 (LD = 38)
	**Triton X-100**	416	37	0.9 (LD = 0.1)	70 (LD = 38)
	**SDS**	-	-	-	-
	**TSPP**	400	37	1.0 (LD = 0.1)	70 (LD = 38)

	**NaCl**	456	39	1.1 (LD = 0.1)	70 (LD = 38)
	**pH 4**	392	36	1.8 (LD = 0.2)	90 (LD = 38)
	**pH 9**	-	-	-	-
**MilliQ**	**Untreated**	204	26		
	**Triton X-100**	196	25		
	**SDS**	140	22		
	**TSPP**	84.0	17		
	**NaCl**	96.0	18		
	**pH 4**	240	28		
	**pH 9**	1100	60		

Table S2. Particle number concentrations, particle recoveries, median mass and median size for extracted Fe NPs in **0.22 µm filtered** samples. Values in brackets represent the limit of detection for the respective measurement. Each PNC and SD represent data from one minute of data collection. Recoveries are calculated relative to the equivalent untreated sample in **Table S1**. Where a "-" is present, there were not a sufficient number of events above the limit of detection.

	**Treatment**	**PNC (particles·L^{-1}, ·10^{7})**	**SD (·10^{7})**	**Recovery (%)**	**Median Mass (fg)**	**Median Size (nm)**
**IAEA-D-8**	**Untreated**	-	-	-		
	**Triton X-100**	181	7	0.025	0.8 (LD = 0.2)	70 (LD = 42)
	**SDS**	200	7	0.031	0.7 (LD = 0.2)	60 (LD = 38)
	**TSPP**	-	-	-		
	**NaCl**	-	-	-		
	**pH 4**	-	-	-		
	**pH 9**	-	-	-		
**IMt-2**	**Untreated**	-	-	-		
	**Triton X-100**	49.6	3.0	0.017	0.6 (LD = 0.2)	60 (LD = 39)
	**SDS**	102	5.0	0.031	0.9 (LD = 0.2)	70 (LD = 40)
	**TSPP**	2110	24	0.97	3 (LD = 0.2)	100 (LD = 40)
	**NaCl**	-	-	-		
	**pH 4**	-	-	-		
	**pH 9**	-	-	-		
**JA-2**	**Untreated**	-	-	-		
	**Triton X-100**	60	4.0	0.056	0.9 (LD = 0.1)	70 (LD = 37)
	**SDS**	364	10	0.54	0.5 (LD = 0.1)	60 (LD = 37)
	**TSPP**	72.8	5.0	0.11	0.5 (LD = 0.2)	60 (LD = 38)
	**NaCl**	-	-	-		
	**pH 4**	-	-	-		
	**pH 9**	54.4	4.0	0.023	0.4 (LD = 0.1)	50 (LD = 37)
**JB-2**	**Untreated**	-	-	-	-	-
	**Triton X-100**	76	5	0.048	1.0 (LD = 0.2)	70 (LD = 41)
	**SDS**	1660	21	1.1	1.0 (LD = 0.2)	70 (LD = 42)
	**TSPP**	1450	20	1.0	1.0 (LD = 0.3)	80 (LD = 46)
	**NaCl**	-	-	-	-	-
	**pH 4**	-	-	-	-	-

	**pH 9**	-	-	-	-	-
**MESS-4**	**Untreated**	-	-	-		
	**Triton X-100**	352	10	0.072	1.3 (LD = 0.3)	80 (LD = 46)
	**SDS**	472	11	0.11	23 (LD = 0.3)	200 (LD = 46)
	**TSPP**	-	-	-		
	**NaCl**	-	-	-		
	**pH 4**	-	-	-		
	**pH 9**	-	-	-		
**R6M-1**	**Untreated**	-	-	-	-	-
	**Triton X-100**	-	-	-	-	-
	**SDS**	-	-	-	-	-
	**TSPP**	-	-	-	-	-
	**NaCl**	-	-	-	-	-
	**pH 4**	-	-	-	-	-
	**pH 9**	-	-	-	-	-
**MilliQ**	**Untreated**	2.80	0.9			
	**Triton X-100**	22.0	2.4			
	**SDS**	10.8	1.7			
	**TSPP**	4.40	1.1			
	**NaCl**	3.20	1.0			
	**pH 4**	14.4	2.0			
	**pH 9**	15.6	2.1			

Table S3. Particle number concentrations, particle recoveries, median mass and median size for extracted Fe NPs in **centrifugated** samples. Values in brackets represent the limit of detection for the respective measurement. Each PNC and SD represent data from one minute of data collection. Recoveries are calculated relative to the equivalent untreated sample in **Table S1**. Where a "-" is present, there were not a sufficient number of events above the limit of detection.

	**Treatment**	**PNC (particles·L^{-1}, ·10^{7})**	**SD (·10^{7})**	**Recovery (%)**	**Median Mass (fg)**	**Median Size (nm)**
**IAEA-D-8**	**Untreated**	83500	1700	11.9	0.6 (LD = 0.1)	60 (LD = 36)
	**Triton X-100**	94000	1800	13.4	0.6 (LD = 0.1)	60 (LD = 36)
	**SDS**	97000	1900	13.8	0.6 (LD = 0.1)	60 (LD = 36)
	**TSPP**	101000	1900	14.4	0.7 (LD = 0.2)	60 (LD = 39)
	**NaCl**	90750	1800	13.0	0.5 (LD = 0.1)	60 (LD = 36)
	**pH 4**	-	-	-	0.6 (LD = 0.1)	60 (LD = 35)
	**pH 9**	97800	1900	14.0	0.6 (LD = 0.1)	60 (LD = 36)
**IMt-2**	**Untreated**	39700	1200	8.9	0.4 (LD = 0.1)	50 (LD = 36)
	**Triton X-100**	24350	940	5.4	0.4 (LD = 0.1)	50 (LD = 35)
	**SDS**	33150	1100	7.4	0.4 (LD = 0.1)	50 (LD = 35)
	**TSPP**	31550	1100	7.1	0.5 (LD = 0.1)	60 (LD = 37)
	**NaCl**	34550	1100	7.7	0.4 (LD = 0.1)	50 (LD = 35)
	**pH 4**	-	-	-	-	-
	**pH 9**	-	-	-	-	-
	**Untreated**	14800	730	15.4	0.5 (LD = 0.1)	60 (LD = 34)

**JA-2**	**Triton X-100**	16050	760	16.7	0.5 (LD = 0.1)	60 (LD = 34)
	**SDS**	10150	610	10.6	0.5 (LD = 0.1)	60 (LD = 34)
	**TSPP**	8500	550	8.8	0.4 (LD = 0.1)	50 (LD = 34)
	**NaCl**	10600	620	11.0	0.4 (LD = 0.1)	50 (LD = 34)
	**pH 4**	-	-	-	-	-
	**pH 9**	8000	540	8.3	0.5 (LD = 0.1)	60 (LD = 34)
**JB-2**	**Untreated**	9050	570	6.794	0.5 (LD = 0.1)	60 (LD = 35)
	**Triton X-100**	6800	495	5.105	0.4 (LD = 0.1)	60 (LD = 35)
	**SDS**	8450	550	6.344	0.4 (LD = 0.1)	60 (LD = 34)
	**TSPP**	6600	485	4.955	0.5 (LD = 0.1)	60 (LD = 35)
	**NaCl**	6250	475	4.692	0.4 (LD = 0.1)	50 (LD = 34)
	**pH 4**	-	-	-	0.6 (LD = 0.1)	60 (LD = 34)
	**pH 9**	-	-	-	0.7 (LD = 0.1)	60 (LD = 35)
**MESS-4**	**Untreated**	86500	1760	19.438	0.6 (LD = 0.1)	60 (LD = 37)
	**Triton X-100**	107550	2000	24.169	0.6 (LD = 0.1)	60 (LD = 37)
	**SDS**	85500	1800	19.213	0.5 (LD = 0.1)	60 (LD = 37)
	**TSPP**	77000	1700	17.303	0.6 (LD = 0.1)	60 (LD = 37)
	**NaCl**	84800	1800	19.056	0.6 (LD = 0.1)	60 (LD = 37)
	**pH 4**	-	-	-		
	**pH 9**	94050	1800	21.135	0.6 (LD = 0.1)	60 (LD = 37)
**R6M-1**	**Untreated**	-	-	-	-	-
	**Triton x100**	-	-	-	-	-
	**SDS**	-	-	-	-	-
	**TSPP**	-	-	-	-	-
	**NaCl**	-	-	-	-	-
	**pH 4**	-	-	-	-	-
	**pH 9**	-	-	-	-	-
**MilliQ**	**Untreated**	20	7			
	**Triton X-100**	68	12			
	**SDS**	20	6.4			
	**TSPP**	16	5.6			
	**NaCl**	12	5.2			
	**pH 4**	52	10			
	**pH 9**	76	13			

Table S4. Particle number concentrations, and median size for Au spikes in **untreated** samples. Values in brackets represent the limit of detection for the respective measurement. Each PNC and SD represent data from one minute of data collection.

	Treatment	PNC (particles·L^{-1}, ·10^7)	SD (·10^7)	Median Size (nm)
**IAEA-D-8**	**Untreated**	2746.8	91	102
	**Triton X-100**	2462	86	103
	**SDS**	2354	84	100
	**TSPP**	2372	84	103

	**NaCl**	2372	84	99
	**pH 4**	2530.8	87	100
	**pH 9**	2327.2	84	100
**IMt-2**	**Untreated**	2530.8	87	101
	**Triton X-100**	1916.4	76	104
	**SDS**	1730.4	72	99
	**TSPP**	2231.2	82	99
	**NaCl**	2722.8	90	99
	**pH 4**	2150	80	100
	**pH 9**	2060	79	99
**JA-2**	**Untreated**	2764.8	91	101
	**Triton X-100**	2625.6	89	101
	**SDS**	1688.4	71	98
	**TSPP**	2264	82	100
	**NaCl**	2830.8	92	99
	**pH 4**	923.2	52	104
	**pH 9**	1949.2	76	100
**MilliQ**	**Untreated**	2132	79	101
	**Triton X-100**	1790	74	100
	**SDS**	1487	67	100
	**TSPP**	2142	81	98
	**NaCl**	2288	83	97
	**pH 4**	537	40	106
	**pH 9**	2460	87	99
**JB-2**	**Untreated**	2506	875.6	100
	**Triton X-100**	2116.8	788.8	104
	**SDS**	1301.6	624.8	100
	**TSPP**	1978.8	762.8	99
	**NaCl**	2542.8	873.2	99
	**pH 4**	1364.4	639.6	103
	**pH 9**	926.8	527.2	102
**MESS-4**	**Untreated**	2273.2	825.6	103
	**Triton X-100**	2892	992.4	103
	**SDS**	1637.2	700.8	103
	**TSPP**	1922.4	759.2	102
	**NaCl**	2234.4	810.4	104
	**pH 4**	2045.2	783.2	104
	**pH 9**	2387.2	846	103
**R6M-1**	**Untreated**	2854.8	925.2	99
	**Triton X-100**	2678	896	103
	**SDS**	1355.6	644	99
	**TSPP**	2328.4	827.2	101
	**NaCl**	2962.8	942.4	99
	**pH 4**	950.8	534	111

	**pH 9**	2971.6	944	100
**RS3_BAM**	**Untreated**	2478.4	853.6	102
	**Triton X-100**	4108	1110	102
	**SDS**	1738	729.2	101
	**TSPP**	1880.4	750.8	100
	**NaCl**	2566.8	877.2	99
	**pH 4**	716.8	463.6	103
	**pH 9**	2310.4	840.8	97

Table S5. Particle number concentrations, recoveries and median size for Au spikes in **0.22 µm filtered** samples. Values in brackets represent the limit of detection for the respective measurement. Each PNC and SD represent data from one minute of data collection. Where a "-" is present, there were not a sufficient number of events above the limit of detection.

	Treatment	PNC (particles·L^{-1}, ·10^7)	SD (·10^7)	Recovery (%)	Median Size (nm)
**IAEA-D-8**	**Untreated**	-	-	-	-
	**Triton X-100**	193	23	7.0	86
	**SDS**	128	19	4.7	89
	**TSPP**	43	11	1.6	94
	**NaCl**	-	-	-	-
	**pH 4**	-	-	-	-
	**pH 9**	-	-	-	-
**IMt-2**	**Untreated**	-	-	-	-
	**Triton X-100**	327	30	13	103
	**SDS**	267	28	11	98
	**TSPP**	196	24	7.7	92
	**NaCl**	-	-	-	-
	**pH 4**	-	-	-	-
	**pH 9**	-	-	-	-
**JA-2**	**Untreated**				
	**Triton X-100**	721	45	26	102
	**SDS**	795	48	29	95
	**TSPP**	698	45	25	93
	**NaCl**				55
	**pH 4**				
	**pH 9**	230	26	8.3	94
**MilliQ**	**Untreated**				
	**Triton X-100**	497	38	23	103
	**SDS**	233	26	11	98
	**TSPP**	400	34	19	92
	**NaCl**				
	**pH 4**				
	**pH 9**	781	47	37	96
**JB-2**	**Untreated**	-	-	-	-
	**Triton X-100**	784	47	31	102
	**SDS**	356	32	14	92

	**TSPP**	598	41	24	94
	**NaCl**	-	-	-	-
	**pH 4**	-	-	-	-
	**pH 9**	-	-	-	-
**MESS-4**	**Untreated**	-	-	-	-
	**Triton X-100**	116	18	5	95
	**SDS**	198	23	9	101
	**TSPP**	326	30	14	93
	**NaCl**	136	20	6	26
	**pH 4**	-	-	-	-
	**pH 9**	-	-	-	-
**R6M-1**	**Untreated**				
	**Triton X-100**	628	42	22	101
	**SDS**	341	31	12	94
	**TSPP**	355	32	12	98
	**NaCl**				58
	**pH 4**				
	**pH 9**	1116	56	39	97
**RS3_BAM**	**Untreated**				
	**Triton X-100**	1164	58	47	102
	**SDS**	366	32	15	98
	**TSPP**	372	33	15	90
	**NaCl**				
	**pH 4**				
	**pH 9**	633	42	26	96

Table S6. Particle number concentrations, recoveries and median size for Au spikes in **centrifugated** samples. Values in brackets represent the limit of detection for the respective measurement. Each PNC and SD represent data from one minute of data collection. Where a "-" is present, there were not a sufficient number of events above the limit of detection.

	**Treatment**	**PNC (particles·L^{-1}, ·10^{7})**	**SD (·10^{7})**	**Recovery (%)**	**Median Size (nm)**
**IAEA-D-8**	**Untreated**	26	2	0.9	88
	**Triton X-100**	70	3	2.5	89
	**SDS**	18	2	0.7	93
	**TSPP**	12	1	0.5	87
	**NaCl**	21	2	0.8	91
	**pH 4**	43	2	1.6	109
	**pH 9**	21	2	0.8	85
**IMt-2**	**Untreated**	14	1	0.6	90
	**Triton X-100**	105	4	4.1	85
	**SDS**	7	1	0.3	89
	**TSPP**	8	1	0.3	90
	**NaCl**	27	2	1.1	91
	**pH 4**	3	1	0.1	109
	**pH 9**	7	1	0.3	97

**JA-2**	**Untreated**	267	6	9.7	85
	**Triton X-100**	120	4	4.4	90
	**SDS**	8	1	0.3	83
	**TSPP**	9	1	0.3	86
	**NaCl**	11	1	0.4	92
	**pH 4**	11	1	0.4	20
	**pH 9**	113	4	4.1	111
**MilliQ**	**Untreated**	257	23	12.0	102
	**Triton X-100**	159	18	7.4	95
	**SDS**	69	12	3.2	95
	**TSPP**	184	20	8.6	98
	**NaCl**	146	18	6.9	99
	**pH 4**	96	14	4.5	103
	**pH 9**	67	12	3.1	91
**JB-2**	**Untreated**	38	2	1.5	91
	**Triton X-100**	68	3	2.7	26
	**SDS**	2	1	0.1	83
	**TSPP**	2	1	0.1	98
	**NaCl**	15	1	0.6	100
	**pH 4**	36	2	1.4	109
	**pH 9**	34	2	1.3	95
**MESS-4**	**Untreated**	51	3	2.2	102
	**Triton X-100**	99	4	4.3	38
	**SDS**	3	6	0.1	91
	**TSPP**	8	1	0.4	85
	**NaCl**	34	2	1.5	89
	**pH 4**	65	3	2.9	100
	**pH 9**	51	3	2.2	92
**R6M-1**	**Untreated**	340	3	11.9	93
	**Triton X-100**	96	1	3.4	27
	**SDS**	21	7	0.7	46
	**TSPP**	87	14	3.1	29
	**NaCl**	238	22	8.3	98
	**pH 4**	0		0.0	
	**pH 9**	102	15	3.6	88
**RS3_BAM**	**Untreated**	33	8	1.3	84
	**Triton X-100**	115	16	4.6	24
	**SDS**	96	14	3.9	108
	**TSPP**	50	10	2.0	60
	**NaCl**	125	16	5.1	93
	**pH 4**	146	6	5.9	26
	**pH 9**	50	10	2.0	83
